# Antioxidant and Antiglycation Effects of Polyphenol Compounds Extracted from Hazelnut Skin on Advanced Glycation End-Products (AGEs) Formation

**DOI:** 10.3390/antiox10030424

**Published:** 2021-03-10

**Authors:** Ludovica Spagnuolo, Susanna Della Posta, Chiara Fanali, Laura Dugo, Laura De Gara

**Affiliations:** Unit of Food Science and Nutrition, Department of Science and Technology for Humans and the Environment, Campus Bio-Medico University of Rome, via Álvaro del Portillo 21, 00128 Rome, Italy; l.spagnuolo@unicampus.it (L.S.); s.dellaposta@unicampus.it (S.D.P.); c.fanali@unicampus.it (C.F.); l.degara@unicampus.it (L.D.G.)

**Keywords:** advanced glycation end products (AGEs), antiglycation, antioxidant, food chain waste/by-products hazelnut skin, polyphenols

## Abstract

The advanced glycation end-products (AGEs) arise from non-enzymatic reactions of sugar with protein side chains, some of which are oxido-reductive in nature. Enhanced production of AGEs plays an important role in the pathogenesis of diabetic complications as well as in natural aging, renal failure, oxidative stress, and chronic inflammation. The aim of this work is to study antiglycation effects of polyphenol compounds extracted by hazelnut skin that represents an example of polyphenols-rich food industry by-product, on AGEs formation. AGEs derived from incubation of bovine serum albumin (BSA) and methylglyoxal (MGO) were characterized by fluorescence. The phenolics identification and total polyphenol content in hazelnut skin extracts were analyzed by HPLC-MS and the Folin–Ciocalteu method, respectively. Antioxidant efficacy was evaluated by monitoring total antioxidant activity to assess the ABTS radical scavenging activity of samples by TEAC assay and oxygen radical absorbance capacity (ORAC) assay, expressed as millimoles of Trolox equivalents per gram of sample. Data here presented suggest that phenolic compounds in hazelnut skin have an inhibitory effect on the BSA-AGEs model in vitro, and this effect is concentration-dependent. The putative role of the hazelnut skin antioxidative properties for hindering AGEs formation is also discussed. Because of AGEs contribution to the pathogenesis of several chronic diseases, foods enriched, or supplements containing natural bioactive molecules able to inhibit their production could be an interesting new strategy for supporting therapeutic approaches with a positive effect on human health.

## 1. Introduction

Nowadays, the positive correlation between consumption of plant food rich in bioactive components and good health is well known. During the last decade, much research focused on the role of plant food in health maintenance and prevention of chronic disease [1,2]. Moreover, food waste products derived from food processing are still quite rich in interesting bioactive compounds, which could be extracted and used to produce nutraceutical supplements and cosmetic products [3]. Hazelnuts represent an interesting source of by-products, producing a big amount of waste material such as leafy covers, skins, and shells. Hazelnuts are typically consumed whole or used as an ingredient in many processed foods. Recently, the study of their composition has gained attention with the aim to add economic value to waste from hazelnut processing [4,5,6,7].

A wide investigation on hazelnut by-products showed their antioxidant activity by different tests, revealing as they could potentially be considered an excellent source of natural antioxidants [4]. The main bioactive molecules in hazelnut by-products are phenolic compounds, a group of chemical substances widely distributed in plant-derived foods. They include phenolic acids, stilbenes, lignans, and flavonoids [5,8]. Phenolic compounds have been reported to have different beneficial effects on human health, such as antioxidant properties, and, as demonstrated in previous studies, also an anti-glycation function [9]. A recently published review reports the inhibitory effects of polyphenols and plant extracts on the formation of advanced glycation end-products (AGEs) [10]. AGEs are heterogeneous compounds that derive from the reaction of reducing sugars with the free amino groups in proteins, nucleic acids, and lipids in a non-enzymatic Maillard-reaction [11]. The first product of glycation is the instable Schiff base adduct which rearranges to form the Amadori product that breaks down to generate some di-carbonyl compounds such as glyoxal (GO), methylglyoxal (MGO), and deoxyglucosones (3-DG) following both oxidative and non-oxidative pathways. In the final step, irreversible colored compounds called AGEs are formed [12]. Di-carbonyl compounds are very reactive products, and they represent a critical step in AGEs generation because they can also generate hydroxyl aldehydes and the corresponding oxidized acid analogues [13]. For example, MGO can react with lysine residues generating the adducts, through a mechanism that involves intermediates like aldimine that can oxidize through a metal-catalyzed process giving deaminated allysine (adipic semialdehyde). This latter further oxidizes to yield 2-amino adipic acid, which indeed accumulates in oxidative-based disorders [14].

Several colorimetric and fluorimetric methods are available to determine parameters that are indicators of AGE production, such as the modification rate of lysine and arginine side chains, fructosamine content, aggregation state of the modified proteins, and AGE-specific fluorescence [15]. AGEs act through different mechanisms, such as: cross-linking extracellular and intracellular proteins that imply alteration in the biochemical and physiological properties of proteins [16]; the binding to their cell surface receptor RAGE leads to the activation of intracellular signaling cascades causing the transcription of genes that have a central role in the phatogenesis of several diseases and the increase in the production of reactive oxygen species (ROS) [17]. 

It is worth noting that the accumulation of glycation adducts during aging is involved in several diseases such as diabetes, renal diseases, and Alzheimer’s [18]. The inhibition of AGE formation by synthetic aminoguanidine (AG) has been documented. However, the treatment with aminoguanidine in type 1 diabetics has caused serious complications [19]. The search for natural AGE inhibitors could thus represent a valid alternative approach. Indeed, the use of plant extracts showed to inhibit the AGE development more effectively than aminoguanidine. Therefore, phenolic compounds could represent a natural source of glycation inhibitors [10], being able to reduce the production of early Maillard reaction products [20,21,22]. *In vivo* and in vitro studies also revealed positive effects of gallic acid on AGE-induced inflammation [23]. Antiglycative effects of different fruit and seed extracts have also been tested, suggesting that green pepper, peach, and promegranate have the highest capability to inhibit AGE formation, while the extract of hazelnut kernels has a more moderate inhibitory capability [24]. However, it is worth noting that hazelnut skin and kernel have different polyphenol content and molecular biodiversity [25,26,27]. Recently published data indicated that roasted hazelnut skin is a richer source of total phenolics and has the highest antioxidant activity, followed by natural and roasted hazelnuts [7]. The skin is a richer source of these bio-active molecules, at least in quantitative terms [5]. Moreover, hazelnut skin is an important by-product of the food industry, if we consider that more than 160,000 tons of hazelnuts are produced annually only by the Ferrero Hazelnut Company (https://www.hazelnutcompany.ferrero.com, accessed on 1 February 2021; Ferrero International S.A. Findel, Luxemburg). 

Worldwide hazelnut production in 2019/2020 was roughly 528,070 tons. Taking into account that roughly 67% of the total fruit weight is comprised of the shell leads to roughly 353,807 tons of hazelnut shells each year [28,29,30]. Hazelnut skin represents 2.5% by weight of the raw material, and it is discarded upon roasting. It has been traditionally used as animal bedding, but a lot of research underlined its potentiality as a source of natural antioxidants and dietary fibers [31].

Indeed, hazelnut skin represents an interesting source of bioactive molecules for nutraceutical or food supplements production, in a circular economy approach. Valorization of products defined as “waste” can contribute to both solving the problem of waste management and disposal, with a considerable economic advantage for companies, other than being greatly environmental-friendly and offering a vast range of molecules of natural origin easily available and eco-sustainable. 

On these bases, the present study aims to investigate the inhibitory effects on AGEs formation of extracts from hazelnut skin. An in vitro model of AGEs formation induced by MGO was set up and evaluated. Qualitative and quantitative phenolic compounds contents and antioxidant capability were also determined for further studying their relationship with AGE-inhibitory activity.

## 2. Materials and Methods

### 2.1. Extraction of Polyphenols from Hazelnut Skins

Hazelnut skins were pulverized mechanically with a blender, and phenolic compounds were extracted according to the procedure reported by Del Rio et al., with some modifications [5]. Two different extraction procedures were applied: one using a 1% (*v/v*) aqueous formic acid solution as an extractive solvent to obtain an aqueous extract and one using methanol/water (75:25, *v/v*) as an extractive solvent to obtain a methanolic extract. 

An amount of 0.5 g of hazelnut skins was added to 5 mL of 1% (*v/v*) aqueous formic acid solution in a 15 mL centrifuge tubes and were extracted for 30 min in an ultrasound bath (Elmasonic S30H, Elma Schmidbauer GmbH, Singen, Germany) at room temperature, frequency of 37 kHz, and heating power of 200 W. The tube was then heated at 70 °C for 1 h and centrifuged for 10 min at 2151 g. The procedure was repeated two times and the extracts were pulled, filtered with a 0.45 μm filter and stored at –20 °C. For the methanolic extract, 0.5 g of hazelnut skins was added to 5 mL of methanol/H2O (75:25, v/v) solution and extracted for 15 min in an ultrasound bath (Elmasonic S30H, Elma Schmidbauer GmbH, Singen, Germany) and vortexed for 15 min. This procedure was repeated twice. Then, the solution was centrifuged for 10 min at 2151 g. The supernatant was filtered with a 0.45 μm filter and stored at –20 °C until analysis. All chemicals and reagents were purchased from Sigma (Sigma-Aldrich, Milan, Italy). 

### 2.2. HPLC-DAD/MS Analysis of Phenolic Compounds

Hazelnut skin extracts were analyzed using a Shimadzu Prominence LC-20A instrument (Shimadzu, Milan, Italy) equipped with two LC-20 AD XR pumps, SIL-10ADvp, CTO-20 AC column oven, and DGU-20 A3 degasser coupled to an SPD-M10Avp PDA detector and a mass spectrometer detector (LCMS-2010, Shimadzu, Tokyo, Japan) equipped with electrospray (ESI) interface. The Shimadzu LC solution Ver. 3.7 software (Shimadzu, Version 3.7) was used to acquire MS data. Separation was performed using a Core Shell column (150 × 4.6 mm I.D., 2.7 μm d.p.) (Merck KGaA, Darmstadt, Germany), with the mobile phase pumped at a flow rate of 1 mL/min. 

The mobile phase was (A) 1% aqueous formic acid and (B) acetonitrile. Both phenolic extracts were separated using the following gradient: t = 0′ 0%B; t = 40′ 30%B; t = 41′ 100%B; t = 48′ 100%B; t = 49′ 0%B; t = 56′ 0%B. The injection volume was 2 μL. Data were acquired using a DAD in the range 210–400 nm and the chromatograms were extracted at 360 nm for a methanolic extract and at 280 nm for aqueous extract. MS-chromatograms were acquired in negative ionization mode, using the following parameters: nebulizing gas flow rate (N2): 1.5 mL min−1; event time: 1 s; mass spectral range: m/z100–800; scan speed: 1000 amu/s; detector voltage: 1.5 kV; interface temperature: 250 °C; CDL temperature: 300 °C; heat block temperature: 300 °C; interface voltage: −3.50 kV; Q-array voltage: 0.0 V; Q-array RF: 150.0 V.

### 2.3. Determination of Total Phenolic Content

The total polyphenols content of hazelnut skin extracts was measured by the Folin–Ciocalteau method. Quantification of total phenolic content in food or biological sample is based on the reaction of phenolic compounds with a colorimetric reagent, which allows measurement in the visible spectrum. The F–C assay relies on the transfer of electrons in alkaline medium from phenolic compounds to phosphomolybdic/phosphotungstic acid complexes to form blue complexes that are determined spectroscopically [32]. The absorbance at 765 nm was measured by microplate reader (Infinite 200 Pro, Tecan, Männedorf, Switzerland) and measures were performed in triplicate.

The aqueous and methanolic extracts, obtained from the procedure previously described, were combined, and dried by rotary evaporator (Eyela, Tokyo, Japan) at 30 °C to remove solvent. Then, the dry matter was resuspended in 5 mL of methanol/H_2_O (50:50; *v/v*) solution and subsequently analyzed. According to the procedure reported in the literature [27], an aliquot of 20 μL of extract or standard compound was mixed with 100 μL of Folin reagent in 1580 μL of methanol/H_2_O (50:50; *v/v*) solution, followed by incubation for 8 min. Then, 300 μL of Na_2_CO_3_ 0,2g/mL solution was added. The absorbance was measured after incubation at room temperature for 2 h, in the dark using a microplate reader (Infinite 200 Pro, Tecan, Italy). The total phenolic content was determined from a standard curve using gallic acid (0−2000 μg/mL) as a standard and expressed as milligrams of gallic acid equivalents per grams of hazelnut fresh weight (mg GAE/g). All chemicals and reagents were purchased from Sigma (Sigma-Aldrich, Milan, Italy).

### 2.4. Determination of Antioxidant Activity

The antioxidant activity of the MeOH/H_2_O extract of hazelnut skin was determined by the TEAC and ORAC assays, according to a previously described method with some modification [33,34].

The Trolox equivalent antioxidant capacity (TEAC) assay is based on the ability of the antioxidant present in a sample to scavenge the radical cation 2,2′-azinobis (3-ethylbenzothiazoline-6-sulfonic acid) (ABTS) by spectrophotometric analysis. The radical cation ABTS˙ was produced by reacting 7 mM ABTS with 2.5 mM potassium persulfate in aqueous phosphate buffer (5 mM, pH = 7,4) solution, stored in the dark at room temperature for 16 h. ABTS^·+^ is a blue-green chromogen with a characteristic absorption at 734 nm with an absorbance of 0.70 ± 0.04 [33].

Briefly, 10 µL of extract solution, gallic acid, or a standard compound were mixed with 190 µL of ABTS^·+^ solution diluted in phosphate buffer (5 mM, pH = 7.4) in a 96-multiwell plate. Antioxidant compounds in the reaction medium capture the free radical with a loss of color and therefore a reduction in absorbance, corresponding quantitatively to the concentration of antioxidants present. The absorbance was monitored after 10 min using a microplate reader (Infinite 200 Pro, Tecan, Männedorf, Switzerland). A calibration curve was prepared with Trolox as a standard (0–700 μM). Results were expressed as mmol Trolox equivalent (TE) per grams of hazelnut fresh weight or acid gallic.

Oxygen radical antioxidant capacity (ORAC) is another method used to estimate the total antioxidant capacity (TAC) of food or natural products based on the inhibition of the peroxylradical-induced oxidation initiated by thermal decomposition of azocompounds such as [2-2′-azobis(2-amidino-propane) dihydrochloride (AAPH)]. Fluorescein (FL) is used as the fluorescent probe.

According to the method described previously [34], 50 μL of FL (78 nM) and 50 μL of sample, blank or standard were placed in a 96-multiwell-plate, which was heated to 37 °C for 15 min and then 25 μL of AAPH (221 mM) were added in each well.

The fluorescence was measured immediately, and fluorescence intensity (excitation wavelength 485 nm and an emission wavelength of 535 nm) measurements were then taken every 5 min to 90 min using a microplate reader (Infinite 200 Pro, Tecan, Männedorf, Switzerland). A calibration curve was prepared with Trolox as a standard (0–50 μM). The ORAC values are expressed as mmol Trolox equivalents (TE) per grams of hazelnut fresh weight or acid gallic. All chemicals and reagents were purchased from Sigma (Sigma-Aldrich, Milan, Italy).

### 2.5. In Vitro Glycation Assay with BSA-MGO

AGE-BSA was prepared by reacting BSA with MGO according to the method described by Starowicz et al. with some modifications [35]. Briefly, BSA (100 mg/mL) and MGO (500 mM) were dissolved separately in PBS 1× (pH 7,4). Then, 2 mL of each sample were prepared by incubating BSA solution (4 mg/mL) with MGO solution at different concentration (20–30–50–100 mM) in PBS 1× (pH 7.4) containing 0.02% NaN_3_ (to prevent microbe development) for 168 h at 37 °C, in the dark. Subsequently, BSA solution at different concentrations (2–4–10–25–50 mg/mL) with MGO (20 mM) were prepared in the same way. BSA solution without the addition of MGO was incubated under the same conditions and used as a control (BSA-non glycated). 

The hazelnut skin extract, a solution of gallic acid or aminoguanidine (25–50–100–200–400–500 μg/mL), was incubated in the dark with 4 mg mL—1 BSA and 20 mM MGO (in phosphate buffer, pH 7.4, with 0.02% sodium azide). For each experimental condition, 2 mL of sample have been prepared and then incubated at 37 °C for 168 h. All chemicals and reagents were purchased from Sigma (Sigma-Aldrich, Milan, Italy) 

### 2.6. Measurement of AGE Fluorescence

The fluorescence of BSA-MGO model system (AGEs) was measured after incubation as described in detail previously using a microplate reader (Infinite 200 Pro, Tecan, Männedorf, Switzerland) at excitation/emission wavelengths 365/440 nm. Additionally, changes in intrinsic protein fluorescence were detected at excitation/emission wavelengths of 280/350 nm [15]. The inhibitory effect of treatment to gallic acid, aminoguanidine or hazelnut extracts was calculated using the following equation:

[%] Inhibition = [1 − (fluorescence intensity of extract/fluorescence intensity of blank)] × 100.(1)

Data are expressed considering IC50 concentrations, defined as the amount of extract or standard compound (μg/mL) required to reduce AGE formation by 50% and were determined by logarithmic regression analyses (*n* = 3) using GraphPad Prism 6 software.

### 2.7. Statistical Analysis

The graphics and all statistical analysis were performed using GraphPad Prism version 6.0. The data were expressed as mean ± standard deviation of three independent experiments, with at least three technical replicates in each experiment. *p*-value, * *p* < 0.05, ** *p* < 0.01, *** *p* < 0.001 were considered statistically significant. The significance of difference was calculated using one-way ANOVA and Tukey as post-test or *t*-test of multiple or two comparison, respectively.

## 3. Results

### 3.1. HPLC-PDA/MS Quali-Quantitative Analysis of Phenolic Compounds in Methanolic and Aqueous Extracts of Hazelnut Skin

**Figure 1:**
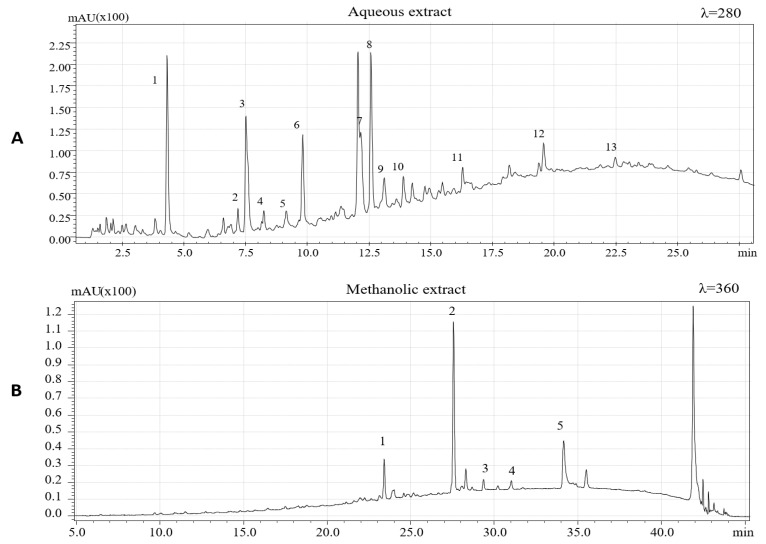
HPLC-PDA chromatograms of hazelnut skin extract: (**A**) aqueous extract (λ = 280); (**B**) methanolic extract (λ = 360). Peak numbers are referred to Table 1 and Table 2.

The chemical characterization of extracted phenolic compounds was carried out by HPLC-PDA/ESI-MS according to experimental conditions previously described. To identify the phenolic compounds, present in the two extracts, the retention time and UV and MS spectra and data available in literature were considered [5,6,7,26,36,37,38,39]. Figure 1 shows HPLC-PDA chromatograms of the aqueous extract (λ = 280 nm) and methanolic extract (λ = 360 nm).

Phenolic compounds identified in aqueous extracts of hazelnut skins are summarized in Table 1. A total of 13 phenolic compounds, being eleven flavan-3-ols and two phenolic acids, were identified based on the mass-to-charge ratio (m/z) of the molecular ion and data available in literature. 

Among flavan-3-ol, (+)-catechin, and (−)- epicatechin, having the same mass-to-charge ratio (m/z), were identified using purified epicatechin as standard molecules and comparing the retention times. These compounds were previously identified in hazelnut skin [5] and are also characteristics of hazelnut shell and kernel as previously reported [6,26,37].

Epicatechin 3-O-gallate, which characterizes the hazelnut shell [6], has been detected with [M−H]^−^ at m/z 441, confirming the results obtained by Del Rio et al. [5]. Among procyanidins, one B-type dimer of procyanidins (PCs) was identified, presenting a [M−H]^−^ at m/z 577. Three procyanidins trimers were detected and one was tentatively identified as procyanidin C2 based on its elution time preceding procyanidin B1. One procyanidin gallate trimer with [M−H]^−^ at m/z 729 and three isomers of B-type prodelphinidin (PDs) dimers were identified based on their [M−H]^−^ at m/z 593. Two isomers of B-type PCs dimers were previously identified in hazelnut shell [6]. Protocatechuic acid ([M−H]^−^ =153) and gallic acid ([M−H]^−^ =169) were also identified. These two hydroxybenzoic acids were previously identified in hazelnut kernels, hazelnut shell, and hazelnut skins [4,5,6,37]. 

In accordance with data reported by Del Rio et al. [5], to extract other less polar flavonoids in hazelnut skin samples, methanolic extraction was performed. Four flavonols and one dihydrochalcone (Table 2) were identified based on their chromatographic and spectrometric behaviors. Three flavonol rhamnosides were identified being myricetin rhamnoside ([M−H]^−^ = 463), quercetin-3-o-rhamnoside ([M−H]^−^ = 447) and kaempferol rhamnoside ([M−H]^−^ = 431). The same compounds were previously detected in hazelnut skin [5] and, except for kaempferol rhamnoside, these compounds have been previously identified also in leaves, shell, and kernels of *C. avellana* L [6,26,37]. One aglycon flavonol, namely, quercetin ([M−H]^−^ = 301), was also detected. This aglycon have previously been identified in hazelnut skin [5] and in hazelnut shells [6]. Finally, a phenolic compound belonging to the dihydrochalcone subclass, namely, phloretin 2-O-glucoside (phloridzin), was also identified. Specifically, this polyphenol showed a [M−H]^−^ at m/z 435. Its presence had already been reported in the hazelnut skin, kernel, and shell [5,6,26]. 

**Figure 2:**
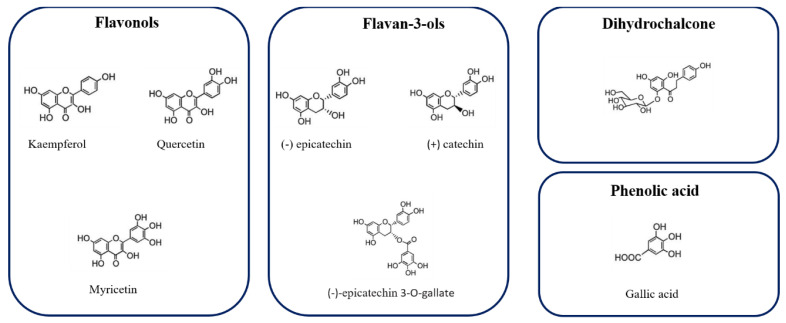
Chemical structure of some phenolic compounds identified in Hazelnut skin extract.

Figure 2 represents the chemical structure of some phenolic compound, identified in Hazelnut skin extract. 

### 3.2. Analysis of Phenol Content and Antioxidant Activity in Hazelnut Skin Extract

Total phenolic content (TPC) and antioxidant activities of hazelnut skin extract were determined by the method previously described. Table 3 shows total phenolic compounds and the values of antioxidant power. Our results indicate that polyphenols in the skin represent about 70 mg GAE/g, (7 g of polyphenol/100 g of hazelnut skin) in line with literature data [5]. This confirms that hazelnut skin could be a relevant polyphenol source compared to the total phenol content (TPC) in hazelnut kernels that represent about 0.07–0.47 mg GAE/g [26]. According to literature data, a mix of flavan-3-ols like procyanidins with their oligomerization forms, (+)-catechin and (−)-epicatechin is the main phenolic component in hazelnut skin (95%). Flavonols and dihydrochalcones represented an additional 3.5% while phenolic acids were responsible for less than 1% of the total identified phenolics in the HPLC-MS/MS method [5]. 

In relation to the antioxidant capability, it is worth noting that, although the antioxidant capability of skin extract was much lower than that of purified gallic acid (Table 3), no remarkable differences were evident between the two methods of analysis used. On the other hand, the antioxidant capability of gallic acid was about four times higher when measured by the ORAC assay than that observed by TEAC assay. Consequently, the difference in antioxidant capacity between skin extract and purified gallic acid remarkably depend on the used methods. This is probably due to the presence in the skin extract of different molecules able to optimize the scavenging properties against the different oxidant species used in the two methods for the determination of antioxidant capacity. Meanwhile, when a purified molecule is used, different sensitivity/specificity in reacting with other chemical species could explain the remarkable difference in its antioxidant capacity. 

In the last few years, accumulating evidence from epidemiological and clinical studies indicates that the daily intake of foods rich in (poly)phenols may possess protective effects in humans [40]. In this context, a positive association between nut, including hazelnut, consumption, and lower risk of all-cause mortality and cardiovascular disease, has been observed [41,42]. Hazelnut skin has been investigated as a functional ingredient rich of bio-active molecules, showing that its addition to coffee, bread, and yogurt improved their physiologically positive effects and antioxidative activity [43,44,45]. The hazelnut skin extracts also revealed functional activity significantly improving the growth of two probiotic strains (Lactobacillus plantarum P17630 and Lactobacillus crispatus P17631), when added in bacterial media [46]. In vivo, hazelnut skin administration in hamsters improved their plasma lipid profile, following a high fat diet [47]. A recent report also shows a potential role for phenolic hazelnut skin extract as a UV protection booster [48].

### 3.3. AGE Quantification

**Figure 3:**
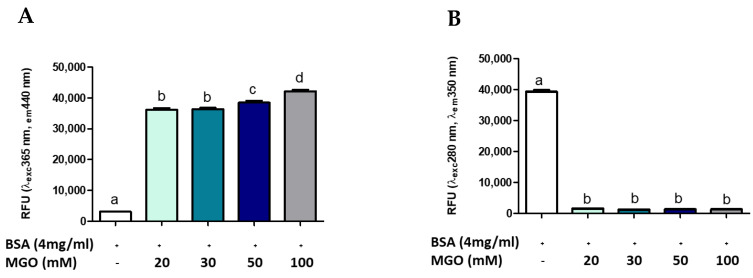
Fluorescence measurement of AGE after 168 h of incubation. All values are expressed as M ± SD (*n* = 3). Data represent relative fluorescence units (**A**) RFU λexc 365 nm/λem 440 nm and (**B**) RFU λexc 280 nm/λem 350 nm of BSA non-glycated (BSA) and BSA glycated (BSA+MGO). Different letters represent significant differences among sample (ANOVA one-way and Tukey post-test).

**Figure 4:**
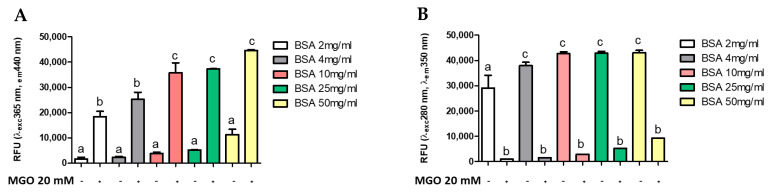
Fluorescence measurement of AGE after 168 h of incubation. All values are expressed as M ± SD (*n* = 3). Data represent relative fluorescence units (**A**) RFU λexc 365 nm/λem 440 nm and (**B**) RFU λexc 280 nm/λem 350 nm of BSA non-glycated (BSA) and BSA glycated (BSA+MGO). Different letters represent significant differences among sample (ANOVA one-way and Tukey post-test).

Several methods are reported in the literature for measuring AGE, many of which use the glycosylation of a standard protein by a standard glycosylation agent [9,15,49]. To evaluate whether AGEs formation was dependent on protein (BSA) or intermediate (MGO) concentration, different concentrations of MGO were incubated with BSA at a standard concentration (4 mg/mL). Figure 3 shows the formation of AGEs during 168 h of glycation reaction. The presence of total AGEs was characterized by fluorescence with respective excitation and maxima emission at 365 and 440 nm. The BSA-MGO model shows a significant formation of fluorescent AGEs, which is already clear with the lowest MGO concentration uses (20 mM). As expected, in the control sample (non-glycated BSA), a very low fluorescence was observed (Figure 3A). To confirm the MGO-dependent BSA glycation, the alterations in the fluorescence of BSA were analyzed: non-glycated BSA showed maximum fluorescence at 280/350 nm, while the 280/350 nm BSA fluorescence intensity strongly decreased as the consequence of MGO treatment (Figure 3B), thus confirming AGEs formation by inducing a conformational change in the protein [15]. AGEs formation with dependence on protein concentration was also confirmed by using a range of 2–50 mg/mL BSA (Figure 4). The comparison between the amount of BSA and BSA-MGO complex at 365/440 nm and 280/350 nm indicates that, at 4 mg/mL, most of BSA was converted in the glycated form, while, at higher BSA concentrations, an increased amount of protein remained in the native form. Therefore, in the following set of experiments, 4 mg/mL BSA was used. 

The comparison of different fluorimetric analysis and timing suggests that, in our conditions (BSA 4mg/mL as standard protein and MGO 20 mM as glycosylated agent), the best time of incubation is seven days, since an incubation time over seven days did not determine a further increase in AGE formation (Figure S1). All fluorescent intensities were measured by a spectrophotometer (Infinite 200 Pro, Tecan, Männedorf, Switzerland).

### 3.4. Inhibitory Effect of Hazelnut Skin Extract on AGEs 

During Maillard reaction, the carbonyl groups of a reducing sugar and free amino acid of proteins form Amadori products and various reactive dicarbonyl species such as 3-DG, GO, and MGO. MGO is the crucial intermediates to favor the formation of AGEs in vitro [50]. 

It is known that antioxidant activity of polyphenols relies on their capability to give electrons or hydrogen ions to free radical molecules as well as to free radicals’ scavenging compounds [51]. Moreover, polyphenols can also react with lysine and arginine residues, thus inhibiting the bond between MGO or GO, two major precursors of AGEs, and free aminoacids [52]. The capability of reducing AGE formation has been reported for several phenolic molecules, which are not present or present only as minor components in hazelnut skin [27]. Ferulic acid inhibited the advanced phase of the glycation [22]; epicatechin, p-coumaric acid, and gallic acid decreased protein carbonyls and AGE formation [49]; protocatechuic acid, dihydroferulic acid, p-coumaric acid, p-hydroxybenzoic acid and salicylic acid showed strong inhibition of AGEs formation, with better results in an oleic acid-BSA system than in a glucose-BSA system [9]. Other compounds like (-)-epigallocatechin-3-gallate (EGCG) [53] and proanthocyanidins [51] inhibited AGEs formation trapping MGO, with different flavonoids with vicinal dihydroxyl groups in the B-rings, such as miricitrin, rhamnetin, quercetin, and fisetin, inhibiting fluorescence AGE development [54]. Indeed, in the BSA-MGO system, quercetin traps MGO directly and then significantly inhibits the formation of AGEs [55].

There are two types of AGEs inhibitors: one is synthetic and the other is a natural compound. Both compounds have been assessed as inhibitors against the development of AGEs. Aminoguanidine (AG) or pimagedine is one well-known synthetic inhibitors of AGEs formation; it was first discovered in clinical trials. It traps reactive carbonyl precursors such as MGO, GO, and 3-DG to non-dangerous products, but this synthetic compound has several side effects [56]. The capability of hazelnut skin extract to inhibit AGE formation was also investigated and compared with that of gallic acid, the efficiency of which inhibiting the in vitro AGE formation is known [49], in addition to that of amminoguanidine.

**Figure 5:**
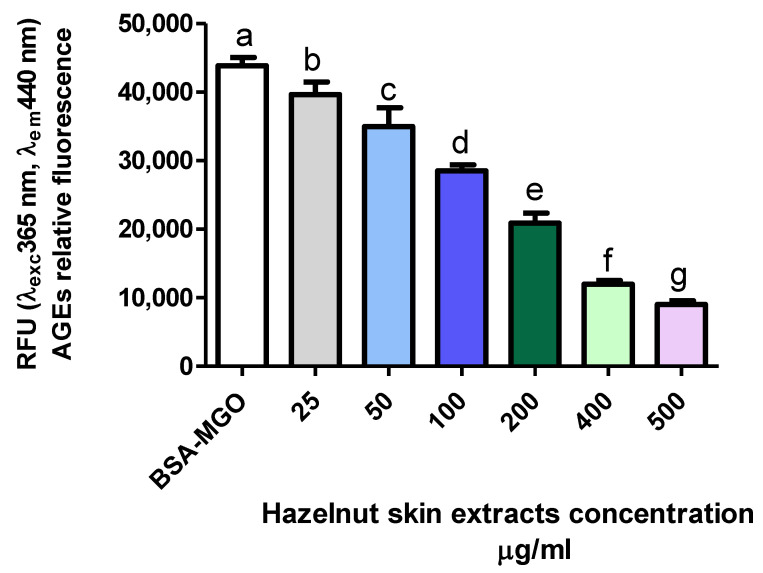
Inhibitory effect on AGE formation. Evaluation of total hazelnut extract (Hz) dose-response effect on AGE formation in vitro. BSA-MGO represents the control group. Different letters represent significant differences among sample (ANOVA one-way and Tukey post-test).

The effect of hazelnut skin extract on the inhibition of AGE formation was dose dependent (Figure 5). Interestingly, hazelnut skin capacity to inhibit AGE formation is slightly and much higher than that of aminoguanidine and gallic acid, respectively, as it is evident by their IC50 (Table 4). The efficiency of a mix of bioactive molecules being higher than a single purified one has been reported for other molecules and biochemical/physio-pathological processes [57]. 

## 4. Conclusions

In this study, we have proven that the (poly)phenolic extract of hazelnut skins can reduce formation of AGEs in vitro. Our data set the basis for further investigation on the effects of hazelnut by-products in the prevention of potentially harmful glycation reactions, although other studies are needed to fully understand how these molecules interact with human physiological and pathological processes. 

AGEs play key roles in cellular response to oxidative stress, through the regulation of different cell signaling pathways. Another class of closely related molecules, the advanced lipoxidation end-products (ALEs), plays alongside to AGEs in the establishment and progression of oxidative stress; elevated production of both ALEs and AGEs leads to protein cross-linking and aggregation, resulting in an alteration of cell signaling and functions, which leads to cell damage and death [58]. 

In this study, we have focused our attention on AGEs alone, although ALEs could represent a topic of interest for future investigation on the potentially healthy effects of bioactive compounds.

In this work, we have shown a potential role for an industrial by-product such as hazelnut skin for the extraction of bioactive compounds; this could represent an advantage for industries that have to deal with rather large amounts of these waste biomasses. The valorization of a waste with nutraceutical potential perfectly fits the circular bio-economy approach, in the ever-growing effort to reduce industrial waste and optimize every step of food production. 

## Figures and Tables

**Table 1:** Peaks identification of hazelnut skin aqueous extract through HPLC/ESI-MS.

N°	Compound Identified	Retention Time (min)	m/z (M−H)^−^
1	Gallic acid	4.43	169
2	Protocatechuic acid	7.32	153
3	Procyanidin C2 trimer	7.55	865
4	Prodelphinidin beta-type dimer	8.19	593
5	Prodelphinidin beta-type dimer	9.05	593
6	Prodelphinidin beta-type dimer	9.59	593
7	Procyanidin beta 1 dimer	11.96	577
8	(+) Catechin	12.41	289
9	Procyanidin beta-type trimer	13.03	865
10	Procyanidin beta-type trimer	13.83	865
11	(-) epicatechin	16.20	289
12	Procyanidin Beta-type dimer gallate	19.55	729

**Table 2:** Peaks identification of hazelnut skin methanolic extract through HPLC/ESI-MS.

N°	Compound Identified	Retention Time (min)	m/z (M−H)^−^
1	Myricetin rhamnoside	23.40	463
2	Quercitin 3-0-rhamnoside	27.52	447
3	Phloretin 2-o-glucoside	29.30	435
4	Kaempferol rhamnoside	30.96	431
5	Quercetin	34.16	301

**Table 3:** Total phenolic content (TPC) and antioxidant capacity (TEAC and ORAC). TPC of Hazelnut skin expressed as mg of gallic acid per g of fresh weight. TEAC and ORAC expressed as mmol of trolox per g of fresh weight. All values are expressed as mean ± SD (*n* = 3).

Compound	TPC (mg GAE/g)	TEAC (mmol TE/g)	ORAC (mmol TE/g)
Hazelnut skin 100 mg/mL	70.07 ± 1.38	0.28 ± 0.03	0.35 ± 0.02
Gallic acid 1 mg/mL		10.98 ± 1.89	42 ± 3.34

**Table 4:** IC50 value of synthetic or natural compounds: total hazelnut extract, gallic acid, and aminoguanidine. The concentration required for a 50% inhibition of the intensity of fluorescence (l = 365/440 nm) was calculated from the dose–inhibition curve, obtained by GraphPad analysis (*n* = 3).

Compounds	IC50 (μg/mL)
Hz extract	109.7
gallic acid	147.6
AG	117.8

## Data Availability

The data presented in this study are available on request from the corresponding author.

## Supplementary Materials

The following are available online at https://www.mdpi.com/2076-3921/10/3/424/s1, Figure S1: Comparison of Fluorescence measurement of AGE at different time.
